# Supplementary material for: Client-care provider interaction during labour and birth as experienced by women: Respect, communication, confidentiality and autonomy
Source: PLoS One. 2021 Feb 12;16(2):e0246697. doi: 10.1371/journal.pone.0246697 (PMC7880498; doi:10.1371/journal.pone.0246697)
Supplement: S1 Table — (DOCX) [file pone.0246697.s001.docx]

**S1 Table. Univariate (OR) and multivariate logistic regression (AOR) models to assess variables associated with optimal interaction in the domains respect and communication.**

| **Characteristics** | | **Respect** | | | **Communication** | | |
| --- | --- | --- | --- | --- | --- | --- | --- |
|  |  | **Optimal interaction n (%)** | **OR [95% CI]** | **AOR [95% CI]** | **Optimal interaction n (%)** | **OR [95% CI]** | **AOR [95% CI]** |
| Total | | 409 (53.3) |  |  | 346 (45.1) |  |  |
| Age | <25 | 21 (70) | *ref** | *ref* | 18 (60) | *ref* | *ref* |
|  | 25-29 | 114 (56.4) | 0.56 [0.24-1.27] | 1.12 [0.30-4.82] | 102 (50.5) | 0.68 [0.31-1.49] | 0.80 [0.22-2.95] |
|  | 30-34 | 178 (52) | 0.47 [0.21-1.05] | 0.49 [0.18-1.32] | 140 (40.9) | 0.46 [0.22-1.00] | 0.44 [0.17-1.15] |
|  | 35-39 | 80 (48.2) | **0.40 [0.17-0.92]** | 0.65 [0.25-1.66] | 71 (42.8) | **0.50 [0.23-1.10]** | 0.44 [0.18-1.08] |
|  | ≥40 | 15 (62.5) | 0.71 [0.23-2.23] | 0.67 [0.26-1.71] | 13 (54.2) | 0.79 [0.27-2.33] | 0.67 [0.27-1.66] |
| Ethnicity | Dutch | 337 (54.4) | *ref* | *ref* | 291 (47) | *ref* | *ref* |
|  | Non-Dutch | 44 (50.6) | 0.86 [0.55-1.34] | 0.87 [0.51-1.47] | 41 (47.1) | 1.01 [0.64-1.58] | 0.91 [0.54-1.54] |
| Education level | Low | 24 (72.7) | *ref* | *ref* | 20 (60.6) | *ref* | *ref* |
|  | Middle | 118 (62.1) | 0.62 [0.27-1.40] | 0.50 [0.21-1.20] | 106 (55.8) | 0.82 [0.39-1.75] | 0.70 [0.31-1.55] |
|  | High | 235 (49.7) | **0.37 [0.17-0.81]** | **0.34 [0.15-0.79]** | 202 (42.7) | **0.49 [0.24-0.99]** | 0.51 [0.24-1.12] |
| Parity | Primiparous | 211 (48.8) | *ref* | *ref* | 175 (40.5) | *ref* | *ref* |
|  | Multiparous | 172 (61.4) | **1.67 [1.23-2.27]** | 1.17 [0.80-1.71] | 159 (56.8) | **1.93 [1.42-2.62]** | **1.49 [1.20-2.17]** |
| Onset of labour | Spontaneous | 331 (55.3) | *ref* | *ref* | 285 (47.6) | *ref* | *ref* |
|  | Induction | 63 (45.3) | **0.67 [0.46-0.97]** | 0.88 [0.51-1.47] | 50 (36) | **0.62 [0.42-0.91]** | 0.71 [0.48-1.13] |
|  | Cesarean section | 15 (51.7) | 0.87 [0.41-1.83] | 2.05 [0.26-16.09] | 11 (37.9) | 0.67 [0.31-1.45] | 0.97 [0.09-10.41] |
| Mode of birth | Spontaneous vaginal birth | 294 (57) | *ref* | *ref* | 261 (50.6) | *ref* | *ref* |
|  | Vaginal birth with episiotomy | 47 (53.4) | 0.87 [0.55-1.36] | 1.23 [0.72-2.08] | 30 (34.1) | **0.51 [0.32-0.81]** | **0.56 [0.33-0.95]** |
|  | Assisted vaginal birth | 30 (42.9) | **0.57 [0.34-0.94]** | 1.05 [0.59-1.88] | 27 (38.6) | 0.61 [0.37-1.02] | 0.99 [0.55-1.78] |
|  | Planned cesarean section | 13 (54.2) | 0.89 [0.39-2.03] | 0.69 [0.07-6.45] | 10 (41.7) | 0.70 [0.30-1.60] | 0.82 [0.70-10.29] |
|  | Unplanned cesarean section | 25 (36.8) | **0.44 [0.26-0.74]** | 0.64 [0.34-1.17] | 18 (26.5) | **0.35 [0.20-0.62]** | **0.50 [0.26-0.96]** |
| Place of birth | At home with community midwife | 129 (75.4) | *ref* | *ref* | 105 (61.4) | *ref* | *ref* |
|  | At the birth center or hospital with community midwife | 79 (58.5) | **0.46 [0.28-0.75]** | **0.53 [0.31-0.90]** | 69 (51.1) | 0.66 [0.42-1.04] | 0.73 [0.44-1.21] |
|  | At the hospital | 200 (43.6) | **0.25 [0.17-0.37]** | **0.31 [0.19-0.51]** | 171 (37.3) | **0.37 [0.26-0.54]** | **0.52 [0.32-0.83]** |

*Reference category
