# Supplementary material for: Client-care provider interaction during labour and birth as experienced by women: Respect, communication, confidentiality and autonomy
Source: PLoS One. 2021 Feb 12;16(2):e0246697. doi: 10.1371/journal.pone.0246697 (PMC7880498; doi:10.1371/journal.pone.0246697)
Supplement: S2 Table — (DOCX) [file pone.0246697.s002.docx]

**S2 Table. Univariate (OR) and multivariate logistic regression (AOR) models to assess variables associated with optimal interaction in the domains confidentiality and autonomy.**

| **Characteristics** | | **Confidentiality** | | | **Autonomy** | | |
| --- | --- | --- | --- | --- | --- | --- | --- |
|  |  | **Optimal interaction n (%)** | **OR [95% CI]** | **AOR [95% CI]** | **Optimal interaction n (%)** | **OR [95% CI]** | **AOR [95% CI]** |
| All respondents | | 491 (64) |  |  | 278 (36.2) |  |  |
| Age | <25 | 20 (66.7) | *Ref** | *ref* | 7 (23.3) | *ref* | *ref* |
|  | 25-29 | 139 (68.8) | 1.1 [0.49-2.49] | 0.80 [0.21-3.03] | 75 (37.1) | 1.94 [0.80-4.74] | 2.43 [0.68-8.69] |
|  | 30-34 | 215 (62.9) | 0.85 [0.38-1.87] | 0.74 [0.27-2.00] | 123 (36) | 1.85 [0.77-4.42] | 1.80 [0.67-4.83] |
|  | 35-39 | 100 (60.2) | 0.76 [0.33-1.72] | 0.92 [0.36-2.39] | 62 (37.3) | 1.96 [0.79-4.83] | 1.66 [0.64-4.25] |
|  | ≥40 | 15 (62.5) | 0.83 [0.27-2.56] | 1.24 [0.48-3.24] | 10 (41.7) | 2.35 [0.73-7.58] | 1.76 [0.69-4.52] |
| Ethnicity | Dutch | 410 (66.2) | *ref* | *ref* | 234 (37.8) | *ref* | *ref* |
|  | Non-Dutch | 55 (63.2) | 0.88 [0.55-1.40] | 1.17 [0.67-2.03] | 31 (35.6) | 0.91 [0.57-1.45] | 0.87 [0.51-1.47] |
| Education level | Low | 26 (78.8) | *ref* | *ref* | 13 (39.4) | *ref* | *ref* |
|  | Middle | 142 (74.7) | 0.80 [0.33-1.95] | 0.78 [0.31-1.94] | 66 (34.7) | 0.82 [0.38-1.75] | 0.74 [0.34-1.63] |
|  | High | 294 (62.2) | 0.44 [0.19-1.04] | 0.53 [0.22-1.28] | 183 (38.7) | 0.97 [0.47-2.00] | 0.89 [0.42-1.90] |
| Parity | Primiparous | 267 (61.8) | *ref* | *ref* | 154 (35.6) | *ref* | *ref* |
|  | Multiparous | 202 (72.1) | **1.60 [1.16-2.22]** | 1.23 [0.86-1.90] | 113 (40.4) | **1.67 [1.23-2.27]** | 0.95 [0.65-1.39] |
| Onset of labour | Spontaneous | 384 (64.1) | *ref* | *ref* | 234 (39.1) | *ref* | *ref* |
|  | Induction | 87 (62.6) | 0.94 [0.64-1.37] | 1.18 [0.74-1.87] | 37 (26.6) | **0.57 [0.38-0.85]** | 0.77 [0.48-1.25] |
|  | Cesarean section | 20 (69) | 1.24 [0.56-2.78] | 1.67 [0.16-17.47] | 7 (24.1) | 0.50 [0.21-1.18] | 0.98 [0.09-10.23] |
| Mode of birth | Spontaneous vaginal birth | 342 (66.5) | *ref* | *ref* | 212 (41.1) | *ref* | *ref* |
|  | Vaginal birth with episiotomy | 50 (56.8) | 0.66 [0.42-1.05] | 0.85 [0.50-1.44] | 22 (25) | **0.48 [0.29-0.80]** | **0.56 [0.33-0.99]** |
|  | Assisted vaginal birth | 37 (52.9) | **0.57 [0.34-0.93]** | 0.72 [0.41-1.29] | 22 (31.4) | 0.66 [0.39-1.12] | 0.73 [0.40-1.35] |
|  | Planned cesarean section | 17 (70.8) | 1.23 [0.50-3.01] | 1.01 [0.80-12.93] | 6 (25) | 0.48 [0.19-1.22] | 0.60 [0.05-7.59] |
|  | Unplanned cesarean section | 44 (64.7) | 0.93 [0.54-1.57] | 1.26 [0.68-2.33] | 16 (23.5) | **0.44 [0.25-0.79]** | 0.54 [0.28-1.04] |
| Place of birth | At home with community midwife | 136 (79.5) | *ref* | *ref* | 80 (46.8) | *ref* | *Ref* |
|  | At the birth center or hospital with community midwife | 85 (63) | **0.44 [0.26-0.73]** | **0.47 [0.27-0.82]** | 57 (42.2) | 0.83 [0.53-1.31] | 0.81 [0.49-1.33] |
|  | At the hospital | 269 (58.6) | **0.36 [0.24-0.55]** | **0.41 [0.24-0.70]** | 141 (30.7) | **0.50 [0.35-0.72]** | **0.61 [0.38-0.98]** |

*Reference category
