# Supplementary material for: Client-care provider interaction during labour and birth as experienced by women: Respect, communication, confidentiality and autonomy
Source: PLoS One. 2021 Feb 12;16(2):e0246697. doi: 10.1371/journal.pone.0246697 (PMC7880498; doi:10.1371/journal.pone.0246697)
Supplement: Attachment — Submitted filename: Response to Reviewers.pdf [file pone.0246697.s004.pdf]

20 January 2021

Manuscript Number: PONE-D-20-27732

Dear Dr Laganà,

We thank you and the reviewers for the comments on our manuscript: *Client-care provider interaction during labour and birth as experienced by women: respect, communication, confidentiality and autonomy* which we submitted for publication in PLOS ONE and for giving us the opportunity to revise the manuscript.

Please find a detailed response to the comments below. We copied the comments of the reviewers and wrote our response in *Italic* next to each comment. We hope you are in agreement with our edits to the original manuscript and look forward to hearing from you.

Yours sincerely, on behalf of all authors,

Marit van der Pijl, MSc

E: m.vanderpijl@amsterdamumc.nl (MP)

T: +31650892910

Detailed response to the editor and the reviewers

**Editor**	
Comment	*Response*
Please ensure that your manuscript meets PLOS ONE's style requirements, including those for file naming.	*All style requirements have been checked and where necessary adjusted according to the PLOS ONE style templates.*
We note that you have stated that you will provide repository information for your data at acceptance. Should your manuscript be accepted for publication, we will hold it until you provide the relevant accession numbers or DOIs necessary to access your data. If you wish to make changes to your Data Availability statement, please describe these changes in your cover letter and we will update your Data Availability statement to reflect the information you provide.	*The dataset has been checked and approved for sharing by our privacy manager. As previously stated, the relevant accession number/DOI will be provided if the manuscript is accepted for publication.*
Please include a copy of Table 5 which you refer to in your text on page 18.	*We apologise that Table 5 was mistakenly mentioned. This table is not part of the manuscript.*<br>*To keep the manuscript comprehensible, we only included a table with variables that had significant outcomes in the multivariable analysis in the manuscript (Table 4) and uploaded two supporting information files with two tables that provide a complete overview of the univariable and multivariable analyses performed for all four domains (S1 Table and S2 Table). Where applicable, we added a reference to the supporting information files in the revised manuscript. For example, see page 19 line 313 to 314.*

**Reviewer 1**	
Comment	*Response*
The Abstract needs to give the country of where the study was undertaken to give the reader immediate context. This also needs to occur in the Introduction section.	*As suggested by the reviewer we added the country in the abstract, see page 2 line 26 and in the introduction, see page 6 line 113 to 114.*
The Abstract states that the domains were dichotomized to undertake the logistic regression. Is this a validated and accepted approach? What cut offs were used to do this? The results suggest it was 3.5 – why was this selected?	*The dichotomisation process is described in the data analysis section:*<br>*''The answer options 'never' (1), 'sometimes' (2), and 'often' (3) were considered as non-optimal interaction and 'always' (4) was considered optimal interaction.''*<br>*See page 11 line 220 to 222.*<br>*This information is not given in the abstract due to the word count. However, to provide some information on the dichotomisation process we added information on the scores in the abstract, see page 2 line 32 to 33.*<br><br>*With the ReproQ measurement tool, multiple ways of analysis can be used. The way scale 1-4 is categorised or dichotomised depends on the purpose of the study.*

	The purpose of the current study was to determine the level of optimal client-care provider interaction. Therefore, 'always' (score 4) was considered as optimal interaction, as this represents women who continuously felt they experienced optimal interaction during labour and birth. Striving for optimal interaction should be the standard, especially in a high quality health care setting such as the Netherlands. *We added this in the data analysis section, see page 11 line 217 to 220. Furthermore, we also added a paragraph covering this point in the strengths and limitations section, see page 28 line 487 to 491.*
Were all women who filled in the survey included. The Abstract states that 767 were included – was that everyone or was a sample selected?	*Fig 1 shows a flowchart of the respondents and the steps that were taken to select the sample of respondents. We see that this is not clearly stated in the abstract, therefore we changed the first sentence of the results in the abstract to:* *''Of the 1367 recruited women, 804 respondents completed the questionnaire and 767 respondents completed enough questions to be included for analysis.''* *See page 2 line 36 to 37.*
The abstract conclusion states that the majority of women experienced high quality care which is correct but it was actually less than two thirds – more than one third of women reported non-optimal interactions which is concerning.	*We agree with the reviewers that the wording we chose does not fully match our findings. We adjusted our conclusion section, see page 30 line 534 to 538. We adjusted the abstract accordingly, see page 2 line 45 to 49.*
Women who could not read Dutch were not included. What do the authors feel would have been the responses if women from migrant and refugee backgrounds who did not speak Dutch would have been? It is likely that the results would have been lower and this might warrant a comment in the Discussion.	*We agree with the reviewer that the results might have been lower when including women from migrant or refugee backgrounds, as previous research reports that these groups experience a higher level of disrespectful and abusive care during childbirth. We discussed the limitations of the questionnaire only being available in Dutch in the strengths and limitations section, see page 29 line 515 to 519. We agree that it is important to specifically mention women with migrant or refugee background, we added this to the section, see page 29 line 520 to 522.*
The respondents were different on almost every characteristic (Table 2). Why was that and what are the implications for the generalizability?	*Research shows certain types of women are more likely to participate in research than others. It is a challenge to include for example migrant women, women with a refugee background, or women with a lower education. This was also the case in our study. Furthermore, as the questionnaire was only available in Dutch, it is possible this affected the inclusion of respondents with a non-Dutch background. These limitations are addressed in the strengths and limitations section of the manuscript, see page 28-29 line 509 to 522.*
I found Table 3 hard to follow. I understand the overall n and % for each domain and the n and % for the Likert scale results (col	*The 53% in table 3 represents the number of respondents who answered 'optimal' to all questions within the domain. Therefore, the overall percentage of*

3). I don't follow Col 4 for the Likert scale responses. This is one n and % from 5 responses? Equally, for example, the overall % of optimal care for the domain (e.g. Respect) was 53% but each of the domain elements below this are 75% and above. How did the 53% get worked out? Maybe I am not reading this correctly but the table needs for clarity. This is the same for all of table 3.	*the domain is lower compared to percentages shown at the separate questions. We described this in the data analysis section, see page 11 line 222 to 228. To make table 3 more clear, the answer options categorised as optimal care are now shown in bold, see page 17-18-19, table 3.*
Some of the writing is hard to follow and needs some editing. For example – women who gave birth with a community midwifery had optimal scores more often than women …… could be rephrased to be: women who gave birth with a community midwifery a high proportion of optimal scores than women ……	*We thank the reviewer for the suggestion and adjusted the revised manuscript accordingly. For example in the discussion section, see page 23 line 364 to 366.*
There are a number of typographical and spelling errors (e.g. line 61 and 72).	*We apologise for the typographical and spelling errors and we adjusted these throughout the revised manuscript.*
PSTD needs to be written out the first time it is used (line 62).	*We adjusted this point according to the suggestion of the reviewer, see page 4 line 63 to 64.*

**Reviewer 2**	
Comment	*Response*
I would suggest checking the use of British and American English in the text and tables (i.e., the use of both cesarean and caesarean).	*We thank you for this remark. We consistently used British English in the revised manuscript. Therefore we replaced cesarean with caesarean and made other adjustments concerning using the right type of English where needed.*
Regarding study limitations, the main concern regards the high risk of recall bias. Questionnaires were sent two months after delivery.	*We agree the timing of the questionnaire may influence the answers given. Previous literature shows a broad time range in which studies on childbirth experience were conducted, without sufficient evidence on the optimal moment of measurement. To address this topic, we added a paragraph in the strengths and limitations section, see page 28 line 495 to 508.*
I would suggest clarifying the source of delivery details, such as the induction of labor and delivery mode. Were details asked the patients or retrieved by medical records?	*All details on labour and birth were provided by the respondents. No information was retrieved from medical records. We clarified this in the data analysis section, see page 10 line 194-195.*
The response rate should be discussed. Are the characteristics of women who did not respond available?	*The response rate is visualised in Fig 1. Unfortunately, we do not have data on the characteristics of the non-responders. We added this in the strengths and limitations section, see page 29 line 510 to 513.*
Episiotomy appears as an important factor. In this regard, I would suggest discussing more, in general, the importance of avoiding routine use of episiotomy favoring a selective use. Refer to: PMID: 31823037	*We thank the reviewer for this suggestion. We added information on episiotomy in the discussion, including the suggested reference, see page 24 line 400-406.*

Unplanned cesarean section is reported as a factor associated with communication score. Were cesarean section complications considered as a factor influencing such association? Cesarean section is major surgery, and associated complications may have influenced this observed association. Refer to: PMID: 30877907; PMID: 28681107	*The reviewer raises an important point about the role of complications in unplanned caesarean sections. The impact of complications and interventions was mentioned in the discussion, see page 25 line 427 to 430.*<br>*Unfortunately, we did not have information from our study population on the occurrence of complications during caesarean section. We added a paragraph on the influence of unplanned caesarean section, see page 26 line 449 to 455.*<br>*However, previous research showed that interaction and communication with caregivers are often more important factors in women's experiences than interventions taking place during labour and birth. We mentioned this in the introduction to highlight the important role of client-care provider interaction in every kind of birth situation or setting. See page 5 line 85-89.*

**Reviewer 3**	
Comment	*Response*
Suggest that authors provide clear definitions of autonomy and disrespect. These terms are being universally applied but are culturally nuanced. Define it in the Dutch context. Having greater precision around these terms is critical to progress. What is mistreatment? What is respect? What is autonomy? I would guess that providers would also have a hard time defining this term – and the misalignment of values is where we do not direct enough of our analysis or critique of the issues related to RMC.	*We thank the reviewer for raising this important point about clarifying the definitions in the Dutch context. We added a definition of disrespect and abuse in the* introduction*, see page 4 line 66 to 68.*<br>*Additionally, we added a paragraph in the introduction to elaborate on what the four domains of interaction entail, including autonomy, see page 6 line 102 to 112.*
Pg3 (ln 65-70) Early on, authors mention the Vedam (2019) study and women of color , younger women and lower SES– however this analysis is not specify if women of color or income level. Suggest citing their broader statistic that is mentioned later in the manuscript (1 in 6 women experience mistreatment)	We agree that the broader statistic of this study is important to mention, therefore we changed the reference to this paper into:<br>*Vedam et al. (2019) found that one in six women experience mistreatment, with women of colour experiencing more disrespectful care by health care providers during labour and birth compared to white women[15].*<br>See page 4 line 70-72.
Pg4 (ln 80-82) authors state: "Although the literature does not provide one clear definition of interaction, it can be described as a 81 process of cognition and action, in which the actions can be physical acts, acts of interplay or contact 82 of verbal or nonverbal communication [19]" Agreed – however a true analysis of any interaction or relationship requires both parties are	*We agree that interaction goes both ways. To cover this, we added the following lines at the end of the discussion:*<br>*''Lastly, the current study only includes the clients perspective on client-care provider interaction. As the interaction between clients and care providers includes two parties, it is important to in future also study the care providers point of view.''*<br>*See page 29 line 529 to 531.*

addressed. Please specify that this only analyses the client perspective and perhaps in the limitation, it can include that analysis of provider perspectives is needed.	
Consider including the history of RMC being rooted in a human rights framework – this grounds the work in a broader discourse.	*We added a paragraph on the Universal Rights of Childbearing Women in the introduction. See page 5 line 79-84.*
Methods: Recommend the word 'tiers' instead of echelons.	*We thank the reviewer for this suggestion. We think both terms are not best suited in this context. Therefore we decided to rephrase the sentence into: ''The Dutch maternity care system is divided in primary midwife-led care and secondary obstetrician-led care''*<br>*See page 7 line 122 to 123.*
Define 'secondary obstetrician-led units for international audience	*Secondary obstetrician-led care is provided in the hospital setting. We described this in the study setting. See page 7 line 125 to 126.*
Pg. 5(ln111-113) Provide rationale for this investment in collaborative practice? Why was this created?	*We adjusted the paragraph on collaborative practice to clarify that the rationale for this investment was to improve the quality of maternity care. See page 7 line 132-137*
Data analysis:<br>Most of my questions in the analysis is how the decision to dichotomize was made? What was the rationale for not including 'often' with 'always'. This would seem a less skewed dichotomization, but authors did not choose this cutoff. Is there precedent for this cut point? Since your significance was found when using this dichotomization technique – it seems valuable for the readers to understand the rationale for this choice.	*With the ReproQ measurement tool, multiple ways of analysis can be used. The way scale 1-4 is categorised or dichotomised depends on the purpose of the study. The purpose of the current study was to determine the level of optimal client-care provider interaction. Therefore, 'always' (score 4) was considered as optimal interaction, as this represents women who continuously felt they experienced optimal interaction during labour and birth. Striving for optimal interaction should be the standard, especially in a high quality health care setting such as the Netherlands. We added this in the data analysis section, see page 11 line 217 to 220. Furthermore, we also added a paragraph covering this point in the strengths and limitations section, see page 28 line 487 to 491.*
Pg9-10 (lns 203-228) is very particular and shows the thinking process around specific questions – can this be condensed?	*We agree with the reviewer that this part is specific and therefore quite long, however, we do feel it is important to be transparent about the choices we made in categorising the answer options. Therefore, after careful consideration, we decided not to change this in the revised manuscript.*
Results: In the demographic table, you characterize clients as Dutch and non-Dutch. I would think Dutch characterizes your citizenship status? So, can't someone be Turkish and Dutch? Are these mutually exclusive? This categorization, as an American, is odd. For example, you can be Dutch-American or Mexican-American. We categorize by race, which has its own problematics. However, I would suggest if we are talking about interaction in care –	*We agree that we should approach this subject with care. It is important to mention that in the questionnaire, the respondent was asked to choose the population group that they felt they most identified with. Therefore, we refer to it as self-identified ethnicity.*<br>*We acknowledge the limitations in this approach, such as that it does not specifically covers race. We added the following section to the strengths and limitations section:*

racism and xenophobia become critical factors. Every country suffers from racism, so my question is what are you trying to distinguish here? I think in this time of the global movement for Black Lives – it is really important that we, as researchers, apply our tools with precision.	*''Furthermore, the variable of self-identified ethnicity did not provide information on the respondents race. As race plays a role in health care provision[62], this needs to be taken into account when interpreting this variable.''*<br>*See page 29 line 513 to 515.*
For each domain, I would suggest adding a definition to each domain. It may seem tedious and unnecessary, but I think it would help readers and set standards for providers who will be the most likely folks reading this work.	*We added a paragraph on the four domains in the introduction section to make clear what each domain entails. See page 6 line 102 to 112.*
Discussion: It seems overall, people in Holland, more often than not, report feeling respected. However, mistreatment is different than respect and that needs to be clarified here. When we start to use and conflate terms it makes the research less reliable. It would be particularly useful to provide a definition of autonomy and refer to bioethical literature on this concept as well.	*We agree with the reviewer that RMC is not just the absence of disrespect and abuse. We added a paragraph in the discussion to clarify this, see page 24 line 390 to 399.*<br>*Respectful Maternity Care encompasses many factors. Therefore we chose to not merely look at respect, but to take a broader view by using four different domains on interaction. We added a paragraph in the introduction to elaborate on what the four domains of interaction entail, including autonomy. See page 6 line 102 to 112.*
It might also be helpful to cite some feminist principles and literature – while returning to the core human rights principles so readers can situation this work in the more critical conversation of why women are not being listened to, respected or are unable to assert their autonomy.	*We agree that feminism is an important aspect around this topic. We added this to the discussion, see page 24 line 393 to 399.*
Strengths/Limitations: as stated above, clear rationale for how women were identified by their ethnic group is needed if we are to understand the deeper cultural roots of disrespect, mistreatment and limitation placed on one's autonomy. The Dutch system is very unique in terms of your positive outcomes; however, the readership is global. Do not assume that there is a clear rationale for your demographic variables – explain this to us and try to apply an anti-racist frame to why women were categorized this way and why there was no analysis of non-Dutch speakers or refugee women – who may experience – high levels of disrespect and mistreatment.	*See our previous response on the Dutch and non-Dutch ethnicity variable.*<br>*Our questionnaire was only available in Dutch. This is a limitation of our study and we addressed this in the strengths and limitations section, see page 29 line 515 to 517. We agree that the results could have been different when including women from for example migrant or refugee backgrounds, as previous research report that these groups experience a higher level of disrespectful and abusive care during childbirth. As we feel is it important to specifically mention women with migrant or refugee background, we added this to the strengths and limitations section, see page 29 line 520 to 522.*
