# Supplementary material for: Client-care provider interaction during labour and birth as experienced by women: Respect, communication, confidentiality and autonomy
Source: PLoS One. 2021 Feb 12;16(2):e0246697. doi: 10.1371/journal.pone.0246697 (PMC7880498; doi:10.1371/journal.pone.0246697)
Supplement: Attachment — Submitted filename: Plos One-Respect in NL Dec 2020.docx [file pone.0246697.s003.docx]

Manuscript: Client-care provider interaction during labour and birth as experienced by women.

Summary: This manuscript share statistical analysis of a web based survey (ReproQ) to analyze people’s experiences of 4 domains known to impact quality of care: respect, autonomy, communication and confidentiality/privacy. This offers further evidence of the how the tenants of respectful maternity care are being experienced/or not experienced by women.

**Intro:**

Good narrative overview of current literature

Suggest that authors provide clear definitions of autonomy and disrespect. These terms are being universally applied but are culturally nuanced. Define it in the Dutch context. Having greater precision around these terms is critical to progress. What is mistreatment? What is respect? What is autonomy? I would guess that providers would also have a hard time defining this term – and the misalignment of values is where we do not direct enough of our analysis or critique of the issues related to RMC.

Pg3 (ln 65-70) Early on, authors mention the Vedam (2019) study and women of color , younger women and lower SES– however this analysis is not specify if women of color or income level. Suggest citing their broader statistic that is mentioned later in the manuscript (1 in 6 women experience mistreatment)

Pg4 (ln 80-82) authors state: “Although the literature does not provide one clear definition of interaction, it can be described as a 81 process of cognition and action, in which the actions can be physical acts, acts of interplay or contact 82 of verbal or nonverbal communication [19]” Agreed – however a true analysis of any interaction or relationship requires both parties are addressed. Please specify that this only analyzes the client perspective and perhaps in the limitation, it can include that analysis of provider perspectives is needed.

Consider including the history of RMC being rooted in a *human rights framework* – this grounds the work in a broader discourse.

**Methods:**

Recommend the word ‘tiers’ instead of echelons.

Define ‘secondary obstetrician-led units for international audience

Pg. 5(ln111-113) Provide rationale for this investment in collaborative practice? Why was this created?

**Data analysis:**

Most of my questions in the analysis is how the decision to dichotomize was made? What was the rationale for not including ‘often’ with ‘always’. This would seem a less skewed dichotomization, but authors did not choose this cutoff. Is there precedent for this cut point? Since your significance was found when using this dichotomization technique – it seems valuable for the readers to understand the rationale for this choice.

Pg9-10 (lns 203-228) is very particular and shows the thinking process around specific questions – can this be condensed?

**Results**

In the demographic table, you characterize clients as Dutch and non-Dutch. I would think Dutch characterizes your citizenship status? So can’t someone be Turkish and Dutch? Are these mutually exclusive? This categorization, as an American, is odd. For example, you can be Dutch-American or Mexican-American. We categorize by race, which has its own problematics. However, I would suggest if we are talking about interaction in care – racism and xenophobia become critical factors. Every country suffers from racism, so my question is what are you trying to distinguish here? I think in this time of the global movement for Black Lives – it is really important that we, as researchers, apply our tools with precision.

For each domain, I would suggest adding a definition to each domain. It may seem tedious and unnecessary,
